# Evaluation of Anthocyanin Profile and Color in Sweet Cherry Wine: Effect of Sinapic Acid and Grape Tannins during Aging

**DOI:** 10.3390/molecules26102923

**Published:** 2021-05-14

**Authors:** Mingyue Li, Xinjie Zhao, Yuxia Sun, Zhen Yang, Guomin Han, Xue Yang

**Affiliations:** 1 Shandong Provincial Key Laboratory of Microbial Engineering, School of Biologic Engineering, Qilu University of Technology (Shandong Academy of Sciences), Jinan 250300, China; 1043118297@stu.qlu.edu.cn (M.L.); zhaoxinjie1177@163.com (X.Z.); gina35@126.com (Z.Y.); hanguomin@nwsuaf.edu.cn (G.H.); 2 Institue of Agro-Food Science and Technology, Shandong Academy of Agricultural Sciences, Jinan 250100, China; sunyuxia1230@163.com

**Keywords:** sweet cherry wine, sinapic acid, grape tannin, UPLC-Q/TOF-MS, anthocyanin analysis, color evaluation

## Abstract

Cherries are rich in bioactive phenolic compounds and are often fermented into cherry wines. The degradation of anthocyanins during storage will cause color deterioration. The study aimed to utilize sinapic acid and grape tannins in cherry wine to maintain a high fraction in the colored forms of anthocyanins, in order to maximize the color intensity, the latter being associated with good product quality. The effects on the anthocyanin profile and on color parameters of copigments, utilizing spectral measurement combined with UPLC-MS quantitative analysis, have been evaluated in sweet cherry wines. The copigmentation effect of sinapic acid and grape tannin was accompanied by the bathochromic shift and the hyperchromic effect, which lead to an increase in color intensity (lower L*, higher a* and b*). During the aging process, sinapic and grape tannin increased the content of pyranoanthocyanins in cherry wine, especially the addition of sinapic acid makes the cherry wine generate 10-syringyl-pyranocyanidin-3-rutinoside. These results demonstrate that sinapic acid is suitable for adding before alcohol fermentation, while grape tannins can be added before aging.

## 1. Introduction

Sweet cherry (*Prunus avium* L.) is one of the fruits with high commercial value because of its rich nutrients and bioactive compounds with additional health benefits [1]. The main anthocyanins identified in sweet cherries are cyanidin-3-rutinoside and cyanidin-3-glucoside, followed by peonidin-3-rutinoside and pelargonidin-3-glucoside [2,3]. Sweet cherries are also rich in phenolic acids, including neochlorogenic acid, chlorogenic acid and coumaric acid [2,4]. Ferulic acid and caffeic acid are also present in cherry fruits [5,6]. Cherries, like grapes, can be used in winemaking. Color is one of the important indicators of cherry wine, which determines its appeal to consumers. The color of cherry wine is affected by the type and concentration of anthocyanins [7]. However, anthocyanins are unstable and are affected by factors such as pH, temperature, light, metal ions, enzymes, oxygen and the copigmentation effect [8,9].

Copigmentation is widely used to improve and stabilize wine color, which shows hyperchromic effect, accompanied by bathochromic shift [10,11]. The interaction between anthocyanin and copigment forms a “sandwich” structure complex, which protects anthocyanins from nucleophilic attack [12]. Blanco-Vega et al. [13] monitored the formation of vitisin-like pyranoanthocyanin and hydroxyphenyl-pyranoanthocyanin in model wine by HPLC-DAD-ESI-MS/MS, using wine fermentation metabolites and hydroxycinnamic acids. Hydroxyphenyl-pyranoanthocyanins and vitisins are important anthocyanin-derived pigments in the wine aging process [14]. They have a higher color intensity and stability in a wider pH range compared with their anthocyanin precursors, which can be explained by the protective effect of the new pyran ring against the nucleophilic attack of water, which hinders the formation of the carbinol base [15,16]. They exist in the form of pyranoflavylium cations in wine, giving the wine color with an orange hue [17,18]. Sun et al. [19] reported that pyranoanthocyanins synthesized by the reaction of malvidin-3-*O*-glucoside with acetone, pyruvate, ferulic acid, caffeic acid and sinapic acid are more efficiently resistant to bleaching by SO_2_. Among them, vitisin A shows the strongest resistance.

Flavan-3-ols, hydroxycinnamic acid and flavonols are considered to be the main copigments in wine [20]. Yawadio and Morita [21] added carboxylic acid to the anthocyanin-rich fraction of black rice, which can enhance the color of the juice during storage and has the highest efficiency for sinapic acid. Ko et al. [22] added sinapic acid to the anthocyanin solution of black soybean and stored at room temperature for 36 h, the remaining anthocyanin content increased significantly with the concentration of sinapic acid. Compared with the disubstituted (caffeic acid and ferulic acid) and the monosubstituted one (p-coumaric acid), the reaction kinetics of the trisubstituted (sinapic acid) hydroxycinnamic acid toward malvidin-3-glucoside has moderate enhancement [13]. Tannins are often added to improve aroma, taste and long-term color stability [23] in grape winemaking. Liu et al. [24] studied the effects of adding five kinds of tannins to the “Cabernet Sauvignon wine” on anthocyanins and color characteristics. The results showed that grape seed tannins were more effective in enhancing the color intensity of wine. Grape tannins are called condensed tannins and are composed of flavan-3-ol subunits, including catechins, epicatechins, epicatechin-gallate and epigallocatechin [25].

In this study, sinapic acid and grape tannin (factor 1) were added to sweet cherry juice before and after alcohol fermentation (factor 2), respectively, during the wine-making process. Then, the color, the composition profile and content of anthocyanin of cherry wine during the 12-month aging stage were investigated. The study aimed to utilize sinapic acid and grape tannins in cherry wine to maintain a high fraction in the colored forms of anthocyanins, in order to maximize the color intensity, the latter being associated with good product quality.

## 2. Materials and Methods

### 2.1. Materials and Chemicals

Cherry juice were made from sweet cherry of “Tieton” grown in Liaocheng, Shangdong Province (China). The soluble solid content of initial cherry juice was 20 Brix, and its pH was 3.

Sinapic acid was purchased from Shanghai Yuanye Bio-Technology Co., Ltd. (Shanghai, China). Grape tannin (TRS-V) was purchased from Shanghai Jatou industrial and commercial Co., Ltd. (Shanghai, China). Cyanidin-3-glucoside chloride (purity >98%, HPLC) was obtained from Meilun Biotechnology Co. (Dalian, China). LC-MS grade acetonitrile was purchased from Merck (Darmstadt, Germany). LC-MS grade formic acid was purchased from Honeywell Fluka (Seelze, Germany). Ultrapure water was prepared by the Millipore Direct-Q 8 UV-R ultrapure water system (Sigma-Aldrich, St. Louis, MO, USA).

### 2.2. Cherry Wines

Fresh cherry juice was packed in 15 fermentation tanks of 2.5 L capacity, divided into 5 groups of different treatments. Each group was processed in triplicate. The details of the five groups were as follows:

- CK: normal winemaking, no copigment added.
- BFSA: 300 mg/L sinapic acid added before alcohol fermentation.
- AFSA: 300 mg/L sinapic acid added after alcohol fermentation.
- BFGT: 300 mg/L grape tannins added before alcohol fermentation.
- AFGT: 300 mg/L grape tannins added after alcohol fermentation.

(CK: control group; BF: before alcohol fermentation; AF: after alcohol fermentation; SA: sinapic acid; GT: grape tannin).

About 0.3 g/L of active dry yeasts EC1118 (Lalvin Co., Fredericia, Denmark) were activated and mixed in each container. The alcoholic fermentation was then allowed at 25 °C for 11 days. After fermentation, the solids were removed, and each group was bottled with SO_2_ at 40 mg/L and aged for 12 months at 10 °C. Cherry wine samples were taken in the 0th, 3rd, 6th and 12th months after bottle aging, respectively, and the samples were stored at −80 °C for UPLC-MS analysis.

### 2.3. Total Monomeric Anthocyanins and Polymeric Color

According to the method of Giusti et al. [26] with some modifications, the content of total monomeric anthocyanins (TAs) was determined by the pH-differential method, and the polymeric color was determined by the bisulfite bleaching method.

The total monomeric anthocyanins were calculated as follows:

Total monomeric anthocyanins (mg/L) = A × DF × MW × 1000/(ε × 1)

A = (A_520nm_ − A_700nm_)_pH1.0_ − (A_520nm_ − A_700nm_)_pH4.5_

where MW is the molecular weight of cyanidin-3-glucoside, ε is the molar absorptivity of cyanidin-3-glucoside and DF is the dilution factor.

The polymeric color was determined as follows:

Polymeric color = [(A_420nm_ − A_700nm_) + (A_520nm_ − A_700nm_)]_SO2_ × DF

where A is the absorbance of the bisulfite-treated sample

### 2.4. Determination of the Copigmentation Effect

The cherry wine was diluted 10 times using a potassium chloride buffer, pH 1.0. The λ_max_ and A_max_ were obtained by scanning the 400 to 700 nm wavelength range (∆λ = 1 nm), with a microplate reader (BioTek Instruments, Inc. Winooski, VT, USA).

The copigmentation (M) value, which indicates the copigmentation effect, was obtained using the following formula [27]:

M = [(A − A_0_)/A_0_] × 100%

where A and A_0_ are the absorbance value at 520 nm of the copigment-treated and control sample, respectively.

### 2.5. Color Evaluation

The absorbance values of undiluted cherry wine samples were measured at 420, 520 and 620 nm, respectively [28].

Color intensity (CI) = A_420_ + A_520_ + A_620_ Hue (H) = A_420_/A_520_.

The color parameters were measured using the CIELAB method. Absorption of undiluted cherry wine samples was measured at 450, 520, 570 and 630 nm, respectively. The L*, a* and b* values were calculated using the formula reported by Han et al. [29].

### 2.6. UPLC-MS Analysis of Cherry Wine Anthocyanins

The detection and identification of anthocyanins in cherry wines were characterized by an ultraperformance liquid chromatography system (ACQUITY, H-Class, Waters Corporation, Milford, MA, USA) equipped with a time-of-flight mass spectrometer (Q-TOF-MS, Impact Ⅱ, Bruker, Germany). Anthocyanin separation was performed using an Acquity BEH C18 column (100 × 2.1 mm i.d., 1.7 µm; Waters Corporation, Milford, MA, USA). All wine samples, which were filtered with 0.22 µm NYLON66 filters, were injected into the column. The mobile phase consisted of 0.1% aqueous formic acid (A) and acetonitrile (B) with the following gradient elution: 0–5 min: 5% B; 5–12 min: 5–20% B; 12–20 min: 20–40% B; 20–20.1 min: 40–100% B; 20.1–25 min: 100% B; 25–25.1 min: 100–5% B; 25.1–30 min: 5%B. The flow rate of the mobile phase was 0.45 mL/min, and the detection wavelength was 520 nm. The ESI in positive ionization mode was used for the identification of anthocyanins. Mass spectrometry conditions were set as follows: end plate offset, 500 V; capillary voltage, 3500 V; nebulizer, 2.0 Bar; dry gas, 8.0 L/min; dry temperature, 220 °C; collision energy, 7.0 eV. The mass ranged from 100 to 1500 *m/z*. Anthocyanins were quantified as cyanidin-3-*O*-glucoside equivalents.

### 2.7. Data Analysis

Statistical analysis was carried out using one-way analysis of variance and Duncan’s multiple range test to determine significant differences (*p* < 0.05) among the means by IBM SPSS statistics 26 (SPSS Inc., Chicago, IL, USA). The results are expressed as a mean ± standard deviation. Multilinear regression (MLR) was analyzed by SPSS. The contents of eight anthocyanins and TAs were independent variables, and the four color parameters (λ_max_, A_max_, CI and H) were dependent variables.

## 3. Results

### 3.1. Effects of Copigments on Total Monomeric Anthocyanins in Cherry Wine

**Figure 1:**
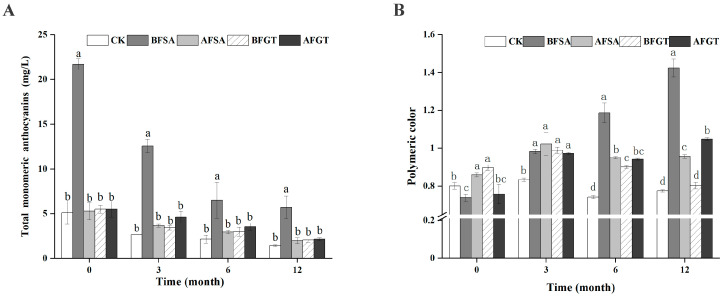
Effects of added sinapic acid and grape tannin on the total monomeric anthocyanins (**A**) and polymeric color (**B**) of cherry wine. Samples with different letters are significantly different (*p* < 0.05).

Adding copigments is an effective way to enhance the stability of natural anthocyanins in a food system by the copigmentation effect [8,30,31]. The changes of total monomeric anthocyanins of cherry wine samples stored at 10 °C are shown in Figure 1A. The total monomeric anthocyanin content of the BFSA group in the aging stage was significantly higher than that of the control group, but the content of the AFSA, BAGT and AFGT groups was not significantly different from that of the control group. The degradation of anthocyanins is always accompanied by the formation of polymeric color, which is consistent with the research of Sinela et al. [32] and Jiang et al. [33]. After 3 months of aging, the copigment also had a significant effect on the polymeric color of cherry wine (*p* < 0.05) (Figure 1B). The increase of polymeric color may be caused by several factors, including condensation, copigmentation or anthocyanin degradation [34,35]. In other words, the increase in polymeric color during the aging process may be because the reaction between anthocyanins themselves or with other phenolic compounds is strengthened by adding copigments.

### 3.2. Evaluation of the Copigmentation Effect of Copigments on Anthocyanins in Cherry Wine

Table 1 shows the changes in the wavelength at maximum absorbance and the maximum absorbance (λ_max_ and A_max_) of cherry wines during one year of aging. Compared with the control, the BFSA group showed an ideal bathochromic shift (5.3 nm) at the beginning of aging. However, BFGT even had a hypsochromic shift of 2.7 nm. After one year of aging, the copigment-treated group showed significant bathochromic shift (*p* < 0.05). Similarly, the four treatments had different degrees of hyperchromic effect. At the beginning of aging, the A_max_ value of BFSA increased by 96%, while the hyperchromic effects of AFSA, BFGT and AFGT were not significantly different from those of the control group. During 3–12 months of aging, the A_max_ of the copigment-treated cherry wine was significantly higher than that of the control group, showing a favorable hyperchromic effect (*p* < 0.05).

The bathochromic shift of cherry wine was related to the formation of pyranoanthocyanin by the copigmentation effect [21]. The pyranoanthocyanin structure increased the chromophore, so it was accompanied by hyperchromic effect. Cherry wine underwent hypsochromic shift and hypochromic effect over time, which may be due to the inevitable degradation of anthocyanins and pyranoanthocyanin during aging.

**Figure 2:**
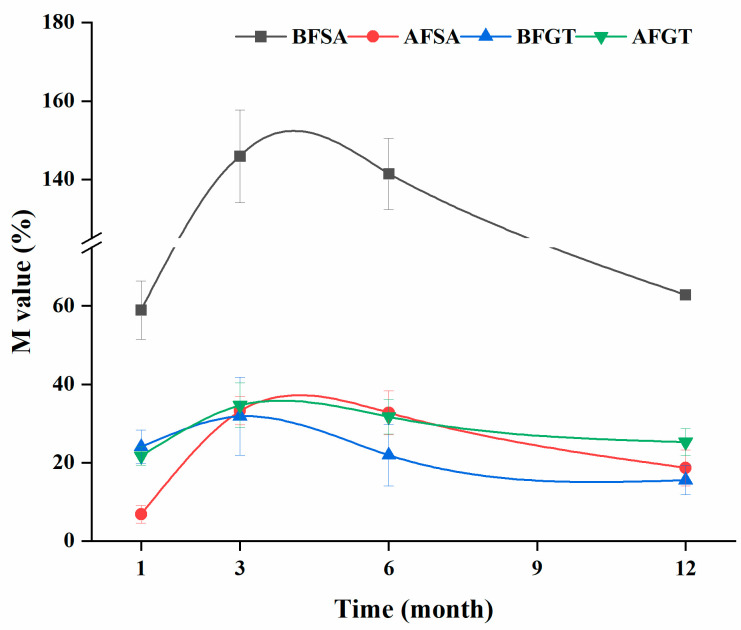
Effects of added sinapic acid and grape tannin on copigmentation effect value (M) of cherry wine.

Figure 2 shows the trend of copigmentation effect on M values, which first increased, then declined and gradually stabilized during aging. Wine samples with pigments reached the highest M value after 3–6 months of aging. Among them, the M value of the BFSA group can reach 146% when aged for 3 months. The concentration of anthocyanins and copigment is an important factor affecting the copigmentation effect [20]. The total anthocyanin content of cherry juice before alcohol fermentation was 31.3 mg/L, while after alcohol fermentation, it decreased to 5.1 mg/L. Zhu et al. [36] investigated that the strongest immediate copigmentation effect at the 1:30 molar ratio occurred between the anthocyanins and sinapic acid. The excellent copigmentation effect of the BFSA group may be contributed by the molar ratio close to 1:30 when 300 mg/L sinapic acid was added before fermentation. Meanwhile, unlike the pyranoanthocyanins derived from coumaric acid and ferulic acid, 10-syringyl-pyranocyanidin-3-rutinoside (derived from sinapic acid) cannot be produced by enzymatic reaction but can be directly produced [37,38]. A large number of anthocyanin monomers were retained before alcohol fermentation, which is conducive to the production of pyranoanthocyanins. Therefore, sinapic acid added before fermentation is more efficient than that added after fermentation.

### 3.3. Evaluation of the Color Effect of Copigments on Anthocyanins in Cherry Wine

**Figure 3:**
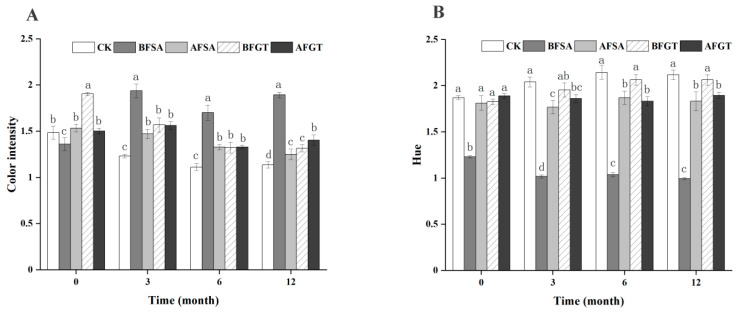
Effects of added sinapic acid and grape tannin on the color intensity (**A**) and hue (**B**) of cherry wine. Samples with different letters are significantly different (*p* < 0.05).

As shown in Figure 3, with the extension of the aging time, the color intensity of the control group decreased and the hue increased, indicating that the color became lighter. The copigmentation effect of sinapic acid, grape tannin and anthocyanin in cherry wine had a positive effect on the color of cherry wine. The copigmentation resulted in a significant increase of color intensity and decreased the hue, indicating a more vivid color characteristics [39]. In the aging stage, the BFSA group maintained a high color intensity, which was related to the hyperchromic effect. The BFGT group showed high color intensity at the beginning of aging. However, due to poor stability, the color intensity decreased with aging, while the AFGT group showed significantly higher color intensity than the control group in the later stage of aging.

**Figure 4:**
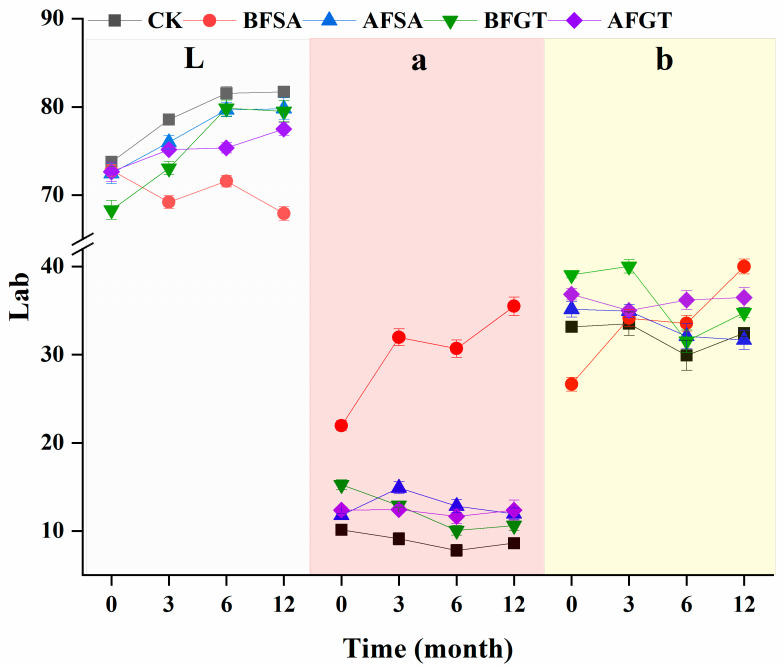
Effects of added sinapic acid and grape tannin on the color parameters brightness (L*), redness (a*) and yellowness (b*) of cherry wine.

In the aging stage, L* of the control group showed an upward trend, while a* and b* showed a downward trend (Figure 4). Compared with the control group, the copigment-treated group showed lower L* values and higher a * and b * values in the CIELAB system, indicating a more pleasurable color characteristic. The lower L* value involves darkness, which may be due to the hyperpigmentation effect caused by copigmentation [24]. Throughout the aging stage, the BFSA group always maintained significantly lower L* values and higher a* values, which were consistent with higher anthocyanin content [36] and presented as a more red–orange color.

### 3.4. Changes in Anthocyanins and Anthocyanin-Derived Compound in the Cherry Wines

**Figure 5:**
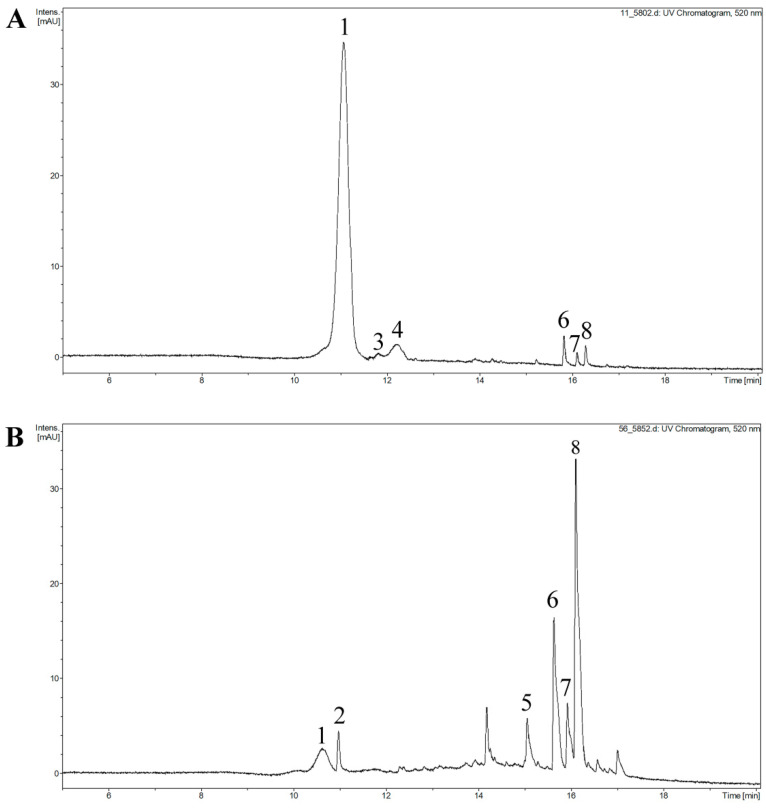
The UPLC chromatograms of BFSA group cherry wine in 520 nm (eight anthocyanins were detected in BFSA group), aging for 0 days (**A**); aging for one year (**B**). Peak 1, cyanidin-3-rutinoside; peak 2, 10-carboxy-pyranocyanidin-3-*O*-rutinoside; peak 3, 10*H*-pyranocyanidin-3-*O*-rutinoside; peak 4, peonidin-3-rutinoside; peak 5, 10-catechypyranocyanidin-3-*O*-rutinoside; peak 6, 10-hydroxyphenyl-pyranocyanidin-3-*O*-rutinoside; peak 7, 10-guaiacyl-pyranocyanidin-3-*O*-rutinoside; peak 8, 10-syringyl-pyranocyanidin-3-rutinoside.

A total of eight anthocyanins were identified by UPLC-Q/TOF-MS, including two monomer forms and six pyranoanthocyanins. The pyranoanthocyanins are divided into two types of vitisins and four types of hydroxyphenyl pyranoanthocyanins. Table 2 lists the detailed information of anthocyanins and anthocyanin derivatives. The UPLC chromatograms and the mass spectra are shown in Figure 5 and Figure S1, respectively. A total of six anthocyanins were detected in the CK, BFGT and AFGT wine samples, seven anthocyanins were detected in the AFSA groups and eight anthocyanins were detected in the BFSA groups.

**Figure 6:**
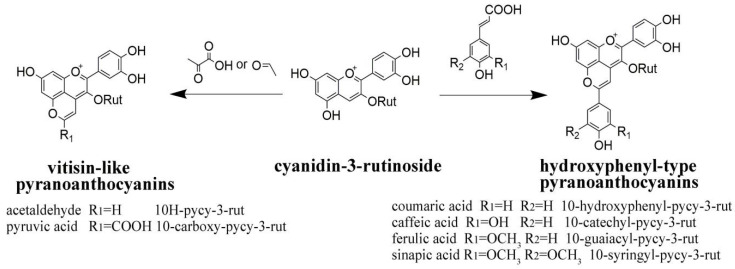
Pathway of formation of pyranoanthocyanins (abbreviations: py = pyrano, cy = cyanidin, rut = 3-O-rutinoside).

**Figure 7:**
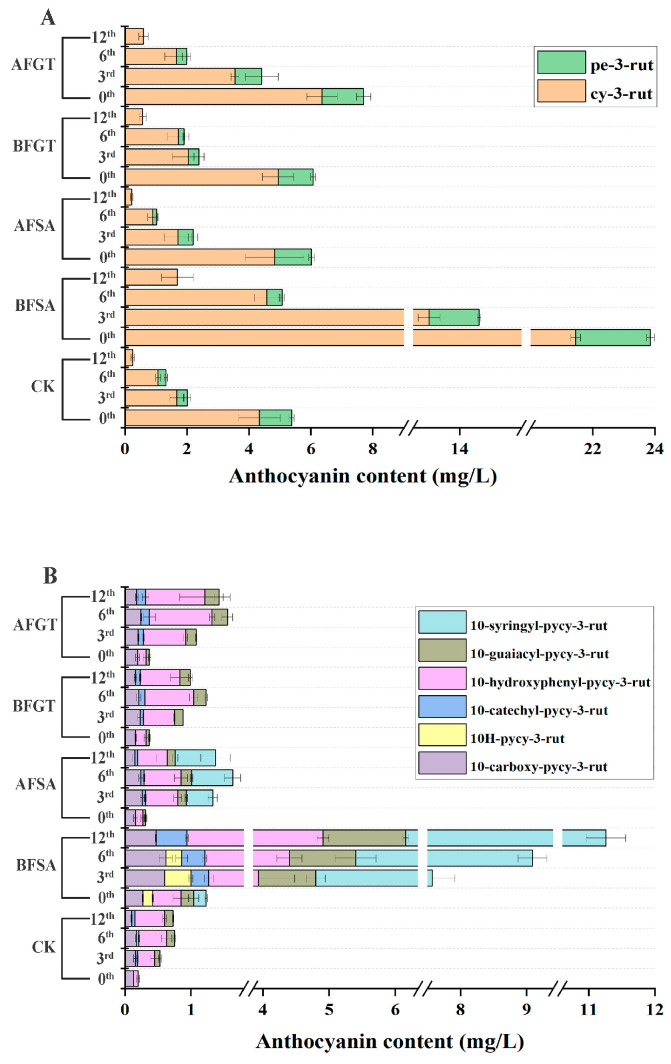
Effects of added sinapic acid and grape tannin on the content of natural anthocyanins (**A**) and pyranoanthocyanins (**B**) during one year aging (py: pyrano; cy: cyanidin; pe: peonidin; rut: 3-*O*-rutinoside; 0th, 3rd, 6th and 12th: the 0th, 3rd, 6th and 12th months of aging, respectively).

Cyanidin-3-*O*-rutinoside (cy-3-rut) was the main anthocyanin in fresh cherry wine, accounting for 76–85% of the total anthocyanin. Peonidin-3-*O*-rutinoside (pe-3-rut) accounted for about 9–18% after alcohol fermentation. Previously, the average concentration of pe-3-rut in cherry wine was about 1.31 mg/L [40], which was consistent with our results. The pyranoanthocyanins in cherry wine are derived from cy-3-rut, and the formation process is shown in Figure 6. It can be seen from Figure 7 that with the extension of aging time, the content of natural anthocyanins gradually decreased, while the content of pyranoanthocyanins gradually increased.

10-Carboxy-pyranocyanidin-3-*O*-rutinoside (10-carboxy-pycy-3-rut) and 10*H*-pyranocyanidin-*3-O*-rutinoside (10H-pycy-3-rut) were formed by reaction of cy-3-rut and yeast metabolites (pyruvic acid and acetaldehyde, respectively). After aging for 6 months, the content of 10-carboxy-pycy-3-rut in the copigment-treated cherry wine was significantly higher than that in control group (*p* < 0.05) and began to decrease slowly during the aging process of 6 to 12 months. This is consistent with the change trend of the pigment in bilberry wine [41]. 10*H*-Pycy-3-rut only appeared in the BFSA group and was not detected at the end of aging. The reason may be that 10H-pycy-3-rut slowly undergoes a hydration reaction, thus causing degradation [17,42].

Peaks 5–8 are four types of hydroxyphenyl pyranoanthocyanins, which were formed by the reaction of cy-3-rut and hydroxycinnamic acid (caffeic acid, coumaric acid, ferulic acid and sinapic acid, respectively) or its decarboxylation products. 10-Catechyl-pyranocyanidin-3-*O*-rutinoside (10-catechyl-pycy-3-rut) was detected after 3 months of aging, 10-hydroxyphenyl-pyranocyanidin-3-*O*-rutinoside (10-hydroxyphenyl-pycy-3-rut) and 10-guaiacyl-pyranocyanidin-3-*O*-rutinoside (10-guaiacyl-pycy-3-rut) could be detected at the beginning of aging. This maybe because pyranoanthocyanins derived from coumaric acid and ferulic acid can not only be formed through cinnamate decarboxylase reactions in the fermentation process but can also be directly formed during aging [38]. The content of pyranoanthocyanins in cherry wine was increased by copigment treatments, in particular, 10-syringyl-pyranocyanidin-3-rutinoside (10-syringyl-pycy-3-rut) only appeared in cherry wine with sinapic acid. Therefore, during the whole aging process, the pyranoanthocyanin content of the BFSA group was significantly higher than that of other groups, followed by the AFSA group and AFGT group. Moreover, it can be seen from Figure 7 that the content of 10-syringol-pycy-3-rut in the BFSA group reached eight times than that of the AFSA group after aging for one year. Three important factors are required for the formation of pyranoanthocyanins [15]. As pyranoanthocyanins are reaction products of anthocyanins, cyanidin-3-rutinoside is necessary for their formation. The second factor is the reaction partner of anthocyanins. Previous studies have demonstrated that hydroxycinnamic acid and yeast metabolites react with anthocyanins to form the corresponding pyranoanthocyanins [19]. The third is storage time. Cherry wine is rich in anthocyanins and possible reaction partners, the formation of pyranoanthocyanins may be formed with increasing storage time, although they may not be present in the initial product. Therefore, some of the most important pyranoanthocyanins found in cherry wine result from the reaction between the original anthocyanins and yeast metabolites released during fermentation, for example pyruvic acid and acetaldehyde. Other pyranoanthocyanins could be obtained by the reaction of anthocyanins with natural phenolic acid present in cherry wines, for example caffeic acid, coumaric acid and ferulic acid. Added copigments belong to phenolic substances, which can promote the extraction of more pigments from the cherry juice into cherry wine, increasing the content of anthocyanins and more phenolic substances [11], thereby, facilitating the conversion of anthocyanins into more stable pyranoanthocyanins.

**Figure 8:**
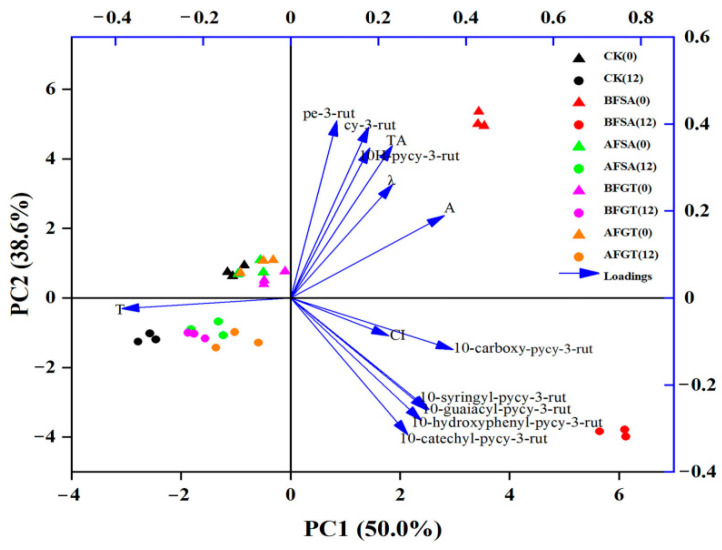
Principal component analysis for cherry wine during aging (0: aging for 0 months; 12: aging for 12 months; arrow: the principal component load; λ: wavelength at maximum absorbance of cherry wine; A: maximum absorbance of cherry wine; CI: color intensity; H: hue; TAs: total monomeric anthocyanins; py: pyrano; cy: cyanidin; pe: peonidin; rut: 3-*O*-rutinoside).

In addition, color parameters (A_max_, λ_max_, CI, H) and anthocyanin content were used for principal component analysis (Figure 8). Based on the analysis results, PC1 represents the red factor (positive) and PC2 the yellow factor (negative). At the beginning of aging, the cherry wine samples were distributed on the upper side of PC2, and after 12 months, they were distributed on the lower side. What is special is that the BFSA group always shows a positive contribution rate to PC1. There are obvious groupings of cherry wines before and after aging, which shows that the cherry wine has changed to a certain extent after aging, mainly due to the decrease of monomer anthocyanins and the increase of pyranoanthocyanins in the cherry wine during the aging process.

The results of MLR analysis (Table 3) showed that pe-3-rut content could explain 83% of color intensity alone and hue was negatively correlated with 10-carboxy-pycy-3-rut and 10-syringyl-pycy-3-rut (*p* < 0.001) and positively correlated with 10-guaiacyl-pycy-3-rut (*p* < 0.05). Furthermore, 10-carboxy-pycy-3-rut, pe-3-rut, 10-syringyl-pycy-3-rut and TAs were statistically positively correlated with A_max_. Among them, A_max_ had the strongest correlation with TAs (*p* < 0.001). The correlation coefficient was 0.678. The pigment with the highest contribution to λ_max_ was 10-carboxy-pycy-3-rut (*p* < 0.05), with correlation coefficients of 0.728. The components and content of anthocyanins had a positive contribution to the color intensity, bathochromic shift and hyperchromic effect of the cherry wine. Our research results indicate that sinapic acid and grape tannin can enrich the components of anthocyanins in cherry wine, so, during the aging process, sinapic acid and grape tannin can improve the color of cherry wine.

### 3.5. UV–Vis Spectra of Anthocyanins and Anthocyanin-Derived Compounds

In the UPLC-MS analysis system, combined with a PDA detector, the UV–vis spectra of anthocyanins and newly formed pyranoanthocyanins were obtained. As shown in Table 2, compared with cy-3-rut, 10-carboxy-pycy-3-rut had a hypsochromic shift (7 nm) of maximum absorption wavelength in the red absorption region (λ_vis-max_). Moreover, the four hydroxyphenyl-type pyranoanthocyanins had a different level of hypsochromic shift. 10-Hydroxyphenyl-pycy-3-rut (one hydroxyl group) and 10-catechyl-pycy-3-rut (two hydroxyl groups) were at 503.3 and 506.7 nm, respectively. 10-Guaiacyl-pycy-3-rut (one methoxy group) and 10-syringyl-pycy-3-rut (two methoxy groups) were at 509.7 and 514 nm, respectively (Figure S2). The hydroxyl and methoxyl groups on the E ring have a positive effect on the stability of the pyranoanthocyanin [19], the higher the hydroxylation or methoxylation level, the greater the λ_vis-max_ value and the higher the color enhancement effect becomes [21]. Pyranoanthocyanins have a greater color expression than anthocyanins at the pH of wine [17], this also explains why the pyranoanthocyanin has a lower maximum absorption wavelength compared to the native anthocyanin but causes the cherry wine to have a bathochromic shift. In addition, anthocyanin is irreversibly degraded during storage, but the content of pyranoanthocyanin increases. This permits to profit from the total coloring capacity of the pyranoanthocyanins at the pH of cherry wine.

## 4. Conclusions

This research studied the changes of color and anthocyanin composition of cherry wine treated with copigments before and after fermentation during aging by spectral measurement combined with UPLC-MS quantitative analysis. The sinapic acid and grape tannin pretreatments of cherry wine exerted positive effects on the content of pyranoanthocyanins and wine color, producing wines with higher color intensity values (lower L*, higher a* and b*) than the traditionally produced wines. Sinapic acid is suitable for adding before alcohol fermentation, while grape tannins can be added after alcohol fermentation. This study provides a strong evidence for the feasibility of the application of copigments in cherry wine.

## Figures and Tables

**Table 1:** Effects of added sinapic acid and grape tannin on λ_max_ and A_max_ of cherry wine.

Time (Month)	Index Tested	CK	BFSA	AFSA	BFGT	AFGT
0	λ_max_	506.67 ± 1.15 bc	512.00 ± 0.00 a	507.33 ± 1.15 b	504.00 ± 2.00 c	506.67 ± 2.31 bc
	A_max_	1.11 ± 0.07 b	2.18 ± 0.04 a	1.13 ± 0.06 b	1.22 ± 0.06 b	1.12 ± 0.05 b
3	λ_max_	500.00 ± 3.00 b	508.00 ± 1.00 a	503.00 ± 2.65 b	500.00 ± 0.00 b	500.33 ± 2.52 b
	A_max_	0.95 ± 0.03 c	1.98 ± 0.07 a	1.07 ± 0.06 b	1.09 ± 0.03 b	1.17 ± 0.06 b
6	λ_max_	501.67 ± 1.53 bc	506.33 ± 0.58 a	502.00 ± 0.00 bc	502.33 ± 2.08 b	499.33 ± 2.08 c
	A_max_	0.86 ± 0.03 d	1.81 ± 0.02 a	1.02 ± 0.01 bc	1.00 ± 0.04 c	1.07 ± 0.04 b
12	λ_max_	493.00 ± 2 c	505.67 ± 0.58 a	500.67 ± 2.89 b	497.67 ± 1.15 b	499.67 ± 3.21 b
	A_max_	0.83 ± 0.02 c	1.78 ± 0.10 a	0.94 ± 0.04 b	0.95 ± 0.02 b	1.02 ± 0.07 b

Note: λ_max_: wavelength at maximum absorbance of cherry wine; A_max_: maximum absorbance of cherry wine. Different letters indicate that there is significant difference between different experimental groups at the same aging time (*p* < 0.05). These data are from the means of values ± standard deviation.

**Table 2:** Anthocyanins and anthocyanin-derived compounds identified in the cherry wines by UPLC-MS.

Peak No.	Compound	RT (min)	[M.H]^+^ (*m/z*)	Fragment (*m/z*)	λ_vis-max_
1	Cyanidin-3-*O*-rutinoside	10.7	595	287	519 ± 0
2	10-Carboxy-pyranocyanidin-3-*O*-rutinoside (vitisin A type)	11.0	663	355	512 ± 0
3	10H-Pyranocyanidin-3-*O*-rutinoside (vitisin B type)	11.8	619	311	nd
4	Peonidin-3-*O*-rutinoside	12.2	609	301	519.67 ± 0.58
5	10-Catechyl-pyranocyanidin-3-*O*-rutinoside	15.1	727	419	506.67 ± 0.94
6	10-Hydroxyphenyl-pyranocyanidin-3-*O*-rutinoside	15.7	711	403	503.33 ± 1.15
7	10-Guaiacyl-pyranocyanidin-3-*O*-rutinoside	16.0	741	433	509.67 ± 0.58
8	10-Syringyl-pyranocyanidin-3-rutinoside	16.2	771	463	514 ± 0

Note: λ_vis-max_: The maximum absorption wavelength of anthocyanins in red absorption region, data are from the means of values ± standard deviation.

**Table 3:** Anthocyanins and anthocyanin-derived compounds identified in the cherry wines by UPLC-MS.

Model	R^2^	DW	F	*P*
CI = 1.397 × pe-3-rut	0.830	0.902	22.277	<0.001
H = −0.424 × 10-carboxy-pycy-3-rut + 0.728 × 10-guaiacyl-pycy-3-rut − 0.698 × 10-syringyl-pycy-3-rut	0.949	1.332	122.371	<0.001
A_max_ = 0.201 × 10-carboxy-pycy + 0.121 × pe-3-rut + 0.180 × 10-syringyl-pycy-3-rut + 0.678 × TA	0.984	1.575	404.704	<0.001
λ_max_ = 0.728 × 10-carboxy-pycy-3-rut − 0.528 × 10H-pycy-3-rut + 0.644 × pe-3-rut	0.746	1.885	16.301	<0.001

Note: R^2^: coefficient of determination; DW: test autocorrelation; F: variance test; P: judge whether the correlation is statistically significant; CI: color intensity; H: hue; λ_max_: wavelength at maximum absorbance of cherry wine; A_max_: maximum absorbance of cherry wine; TAs: total monomeric anthocyanins; py: pyrano; cy: cyanidin; pe: peonidin; rut: 3-*O*-rutinoside.

## Data Availability

The data presented in this study are available in article and Supplementary Material.

## Supplementary Materials

The following are available online, Figure S1: Mass spectra and fragmentation pattern of anthocyanins and anthocyanin-derived compounds in cherry wine. A–H correspond to peaks 1–8 in Figure 5 in turn. Figure S2: The chemical structure of cyanidin-3-rutinoside-derived pyranoanthocyanins in the cherry wine. Reaction products of cyanidin-3-rutinoside (1) with acetaldehyde (2), pyruvate (3), coumaric acid (4), caffeic acid (5), ferulic acid (6) and sinapic acid (7).

## Sample Availability

Samples of the compounds are not available from the authors.
