# Supplementary figures and images for: Evaluation of Anthocyanin Profile and Color in Sweet Cherry Wine: Effect of Sinapic Acid and Grape Tannins during Aging
Source: Molecules. 2021 May 14;26(10):2923. doi: 10.3390/molecules26102923 (PMC8157077; doi:10.3390/molecules26102923)

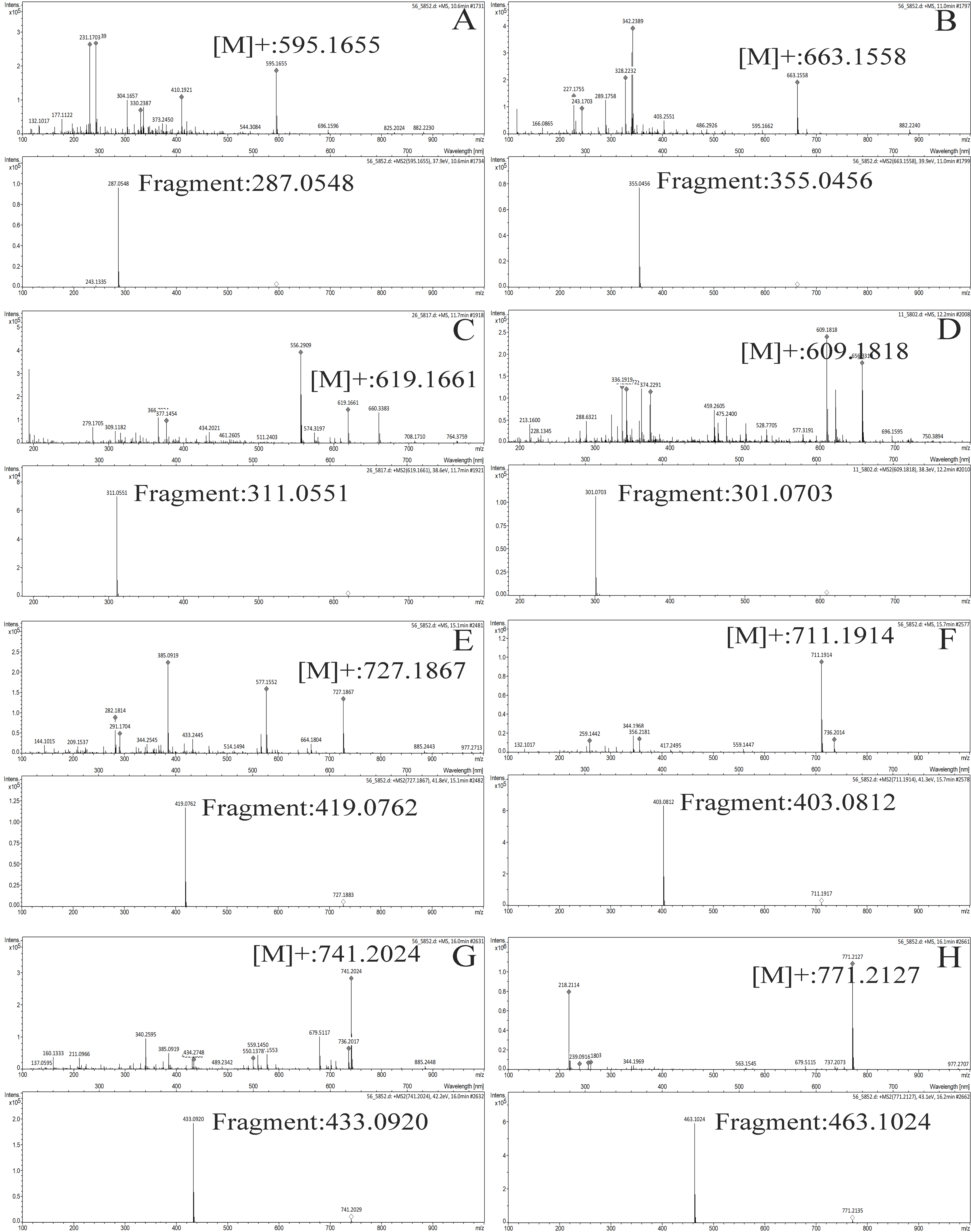

Supplement: Supplementary file 1 [file molecules-26-02923-s001.zip › Supplementary Materials/Fig.S1.tif]
